# Integrating conformal prediction with machine learning for uncertainty-aware risk stratification in coronary artery disease

**DOI:** 10.3389/fcvm.2026.1857390

**Published:** 2026-09-03

**Authors:** Shengnan Lin, Xuyang Hu, Yang Zhang, Qi Zhang, Xiaoning Wang, Yang Yang, Fuzheng Song, Yutong Li, Yushan Liu, Yuanpeng Zhao, Dingjian Zhao, Minghua Nan, Changchuan Bai

**Affiliations:** 1 Department of Cardiology, Liaoning University of Traditional Chinese Medicine, Shenyang, Liaoning, China; 2 College of Integrated Traditional Chinese and Western Medicine, Dalian Medical University, Dalian, Liaoning, China; 3 Department of Out-Patient, Dalian Baichuan Traditional Chinese Medicine Hospital, Dalian, Liaoning, China; 4 Department of Cardiology, The Second Affiliated Hospital of Liaoning University of Traditional Chinese Medicine, Shenyang, Liaoning, China; 5 Specialist Outpatient Clinic, Dalian Hospital of Traditional Chinese Medicine, Dalian, Liaoning, China

**Keywords:** conformal prediction, coronary artery disease, machine learning, risk stratification, uncertainty quantification

## Abstract

**Background:**

Predictive models are increasingly used in clinical decision-making in coronary artery disease (CAD). However, most existing models focus on discriminative ability while ignoring individual prediction uncertainty, which is particularly prominent in small-sample contexts, limiting clinical applications.

**Methods:**

We developed an uncertainty-aware model integrating machine learning and conformal prediction (CP) for CAD prediction in a small-sample setting cohort (*N* = 297). Logistic regression (LR) and random forest (RF) models were trained using clinical variables and evaluated for predictive performance. CP was applied to quantify prediction uncertainty and generate prediction sets with statistical coverage guarantees. Model reliability was assessed using coverage and prediction set characteristics. By combining predicted risk probabilities with uncertainty information, the model transforms traditional binary classification into a four-class framework that reflects both the level of risk (high vs. low) and the certainty of the prediction (high vs. low), thereby enabling a more informative risk stratification.

**Results:**

Both LR and RF show strong discrimination (AUC 0.953 and 0.951). LR is slightly better calibrated, while RF is more accurate. CP efficiently quantifies uncertainty. Union and intersection achieve higher coverage, whereas MSCP with voting balances coverage and efficiency. The combined RF–MSCP model maintains empirical coverage above the nominal level across all significance thresholds, demonstrating robust uncertainty assessment and risk prediction in small-sample settings.

**Conclusion:**

The integration of CP with machine learning has led to the development of the RF-MSCP model, which provides reliable uncertainty quantification for CAD risk prediction, especially in small sample settings. By identifying potentially high-risk individuals overlooked by traditional models, this approach improves predictive discrimination and supports more robust clinical decision.

## 1 Introduction

Coronary artery disease (CAD) is one of the leading causes of death worldwide, accounting for over 19.2 million deaths annually, and is one of the most common and burdensome types of cardiovascular disease (1). With an ageing population and changes in lifestyle, the incidence of CAD continues to rise (2, 3). advances in clinical management, CAD is often characterised by insidious onset and slow progression (4). Consequently, the early identification of high-risk individuals is of critical importance for timely intervention, optimisation of treatment strategies, and improvement of clinical outcomes (4).

In recent years, numerous traditional predictive models have been developed and applied in clinical CAD practice, such as the Framingham Risk Score, the SCORE model, and the ChinaPAR model for assessing the risk of atherosclerotic cardiovascular disease (5–7). these models provide a practical framework for risk estimation, their predictive performance is often subject to various limitations in practical applications, such as inconsistent discriminatory power across different risk populations and population-specific biases, thereby reducing their generalisation ability across different clinical settings. Moreover, these models are usually based on linear assumptions, but there are complex interactions between clinical variables that are difficult to generalise in terms of linear relationships, which limits their potential for application in real clinical scenarios (8).

As an emerging technological tool for predicting CAD risk, machine learning methods have emerged as a research frontier. Compared to traditional models, machine learning can integrate data from multiple sources (e.g., clinical metrics, biomarkers, and images) and construct complex nonlinear relationships, which can significantly improve prediction performance (9–11). Studies have shown that machine learning models exhibit superior discriminative abilities in predicting CAD (12, 13). Recent studies have demonstrated the utility of machine learning approaches in identifying risk factors and improving risk stratification for coronary artery disease (14, 15). However, despite the good discriminative performance of these models, their application to routine clinical practice remains limited (16). Existing studies have mainly focused on discrimination metrics, particularly the area under the receiver operating characteristic curve (AUC) (17). In contrast, other important aspects of model performance, such as calibration and uncertainty quantification, have received far less attention (18). Accurate probability estimates are critical for clinical decision-making, as incorrectly calibrated predictions can lead to inappropriate treatment strategies (19). Traditional machine learning models typically provide only point estimates and struggle to present the uncertainty that accompanies predictions, which may erode clinician trust and hinder their application in real clinical settings (20, 21).

To address these limitations, uncertainty quantification is increasingly becoming an important part of constructing reliable clinical prediction models (22). Recent efforts have explored approaches such as Bayesian inference, Monte Carlo dropout, and ensemble techniques to characterize predictive uncertainty (23–25). However, these methods usually rely on distributional assumptions or are based on large datasets, limiting their widespread use in clinical practice. Conformal prediction (CP) provides an alternative framework that does not require explicit assumptions about the underlying data distribution and offers finite-sample validity under the exchangeability assumptionConformal prediction (CP) is characterised by the fact that it does not rely on distributional assumptions about the data, and CP is capable of reliable uncertainty estimation even with limited samples (26). Predictive probability and predictive uncertainty represent different dimensions of model output. While probability-based risk estimation describes the likelihood of disease occurrence, it does not quantify whether an individual prediction is sufficiently reliable (27). CP complements traditional risk stratification methods by providing an additional measure of predictive confidence at a predefined error level (28). The method allows for direct quantification of forecast uncertainty, thereby improving the interpretability and robustness of the model framework (29). In a binary classification task, the CP prediction set may contain a single label (indicating high certainty) or two possible labels (indicating uncertainty) (30, 31). By combining the predicted risk probability with the size of the prediction set, the risk assessment can be naturally extended into a two-dimensional framework that reflects both the level of risk (high vs. low) and the predicted certainty (high vs. low) (31). This results in four clinically interpretable categories: high risk with high certainty, high risk with low certainty, low risk with high certainty, and low risk with low certainty. The framework not only retains traditional risk information, but also highlights uncertain or borderline cases that may require further clinical assessment (32). Recent studies have shown that the CP method has promising applications in several areas, such as medical image analysis, disease diagnosis, and risk prediction, and can be effective in improving the reliability of clinical decision support systems (33, 34). For example, CP has been used to provide predictions with confidence guarantees in the prediction of multiple sclerosis and in cervical cancer tumour classification and diagnostic imaging (31, 35). Despite these advances, its systematic application in CAD risk prediction remains notably underexplored.

Accordingly, this study aims to construct an uncertainty-aware CAD risk prediction framework by combining CP with machine learning models and constructing models. The approach aims to maximise discriminative performance and provide additional information on the reliability of predictions at the individual level. By combining risk assessment with uncertainty quantification, the proposed model aims to support finer risk stratification and clinical decision-making, exploring its feasibility and potential clinical utility.

## 2 Methods

### 2.1 Dataset and study design

To assess the risk prediction of coronary artery disease with reliability-aware individual decision-making in a limited sample. The baseline coronary artery disease data with moderate dimensionality and limited sample size, the Cleveland subset of the UCI Heart Disease dataset (https://archive.ics.uci.edu/dataset/45/heart + disease), was selected. It's an open cohort containing 303 individuals with coronary clinical assessments. Thirteen clinical predictors together with one outcome variable are summarized (Supplementary Table 1). All variables were numerically encoded to ensure compatibility with machine learning algorithms. The original multi-class diagnostic labels were transformed into a binary outcome, defining positive cases as target > 0 and negative cases as target = 0. Two variables are considered: ca and thal, both of which contain a small number of missing entries marked as “?”. Given the extremely low proportion of missingness, a complete-case analysis was adopted to preserve interpretability and avoid potential distributional distortion that could arise from imputation during subsequent conformal calibration. After excluding incomplete records, 297 subjects remained for analysis. The resulting class distribution was approximately balanced, with 46.13% positive and 53.87% negative cases.

A fixed hold-out evaluation scheme was used for baseline model assessment. The dataset was first divided into a training–calibration pool (80%) and an independent test set (20%). The training and calibration sets were then randomly separated in 3:1. Among them, the independent test set was only used for final model performance evaluation. In the training-calibration pool, an internal split was performed according to the studied conformal strategy: 75% was used for model training and 25% for CP calibration to compute nonconformity scores. All preprocessing steps, including feature standardization, were performed using training data only and subsequently applied to the test set. All thresholds were strictly derived from the calibration set, and this separation was crucial to maintain the limited sample validity guarantee of the CPs. In addition, the preprocessing steps, including feature standardization, were performed using training data only and subsequently applied to the test set.

### 2.2 Baseline probabilistic modeling

Two commonly used models were employed as baseline models for generating individual-level risk estimates for discrimination, calibration, and reliability analyses: logistic regression (LR) and random forest (RF). The LR model is trained using maximum likelihood estimation and input features are standardised prior to model fitting (36). The RF model consists of a set of decision trees and incorporates class weights to mitigate potential class imbalances (37). For the RF model, hyperparameters were determined based on performance in the training data using a grid search strategy. The final parameter settings were: n_estimators = 300, max_depth = 10, min_samples_leaf = 1, max_features = “auto”, and class_weight = ‘balanced' (38, 39).

Baseline model performance was assessed on a test set using traditional discrimination and calibration metrics, with key performance metrics including Accuracy ACC, AUC, Sensitivity and Specificity. ROC and confusion matrices, calibration curves are also plotted to visualise the classification performance. All hyper-parameters were pre-defined in the training data and no model tuning was performed on the independent test set. Both models predicted the positive class probability for each individual, which was subsequently used for performance evaluation and construction of the CP set. Calibration performance was further assessed using the Brier score and expected calibration error (ECE), along with calibration curves for visualisation. These metrics quantify the agreement between predicted probabilities and observed outcome frequencies at the aggregate level (40, 41).

### 2.3 Conformal prediction framework

In clinical decision-making, simply knowing the predicted risk is often insufficient; clinicians also need to understand the reliability of the prediction for a given patient. CP is used to quantify prediction uncertainty, thereby enabling the assessment of prediction reliability for new patients. This process requires no modification to the underlying predictive model and can be applied to any model that outputs probabilities. CP generates a prediction set Γ(x) for each sample, comprising all outcome labels considered plausible at a predefined confidence level (42, 43).

For a given miscoverage rate α∈(0,1), the prediction set Γ(x) is constructed to satisfy the following coverage guarantee: P(Y∈Γ(X))≥1−α.

Within this framework, the inconsistency score is defined as quantifying the inconsistency between model predictions and true labels [44]. Lower predicted probabilities for the true class correspond to higher nonconformity scores. A trained probabilistic model f^(x) was used to estimate class probabilities. A separate calibration dataset $\{\left(X_{i},Y_{i}\right){\}}_{i=1}^{n}$, independent from model training, was used to compute nonconformity scores:

$$s_{i}=1-\hat{\;f}(X_{i}{)}_{Y_{i}}$$

where $\hat{\;f}(X_{i}{)}_{Y_{i}}$ denotes the predicted probability assigned to the true class Y_i_. Nonconformity scores were computed for all samples in the calibration set. Given a specified miscoverage level *α*, the empirical (1 − *α*) quantile of these scores was used as the threshold q*_α_*. For a new input *x*, the prediction set Γ(x) was defined as:

$$\Gamma (x)=\;y:1-P_{y}(x)\leq q_{\alpha }$$

In this study, the formula sets the error coverage rate *α* = 0.10, which corresponds to a coverage rate of 90%.

### 2.4 Reliability and stability assessment

To assess the stability and reliability of the CP results, two conformal strategies were compared: multi split conformal prediction (MSCP) and cross-conformal prediction (CCP) (45, 46). MSCP divides the training-calibration pool was randomly divided into a model training subset (75%) and a calibration subset (25%). Models were fitted using the training subset and the calibration subset computed nonconformity scores to construct the prediction set. The procedure was repeated 15 times to reduce the variability associated with a single random assignment. The repeatedly split prediction set was aggregated by three strategies: voting, union, and intersection. In the voting strategy, category labels were included in the final prediction set if they appeared in the majority of repetitions. In the joint strategy, the prediction set contains all labels that appear in any repetition in the repetitions. In the intersection strategy, only labels appearing in each repetition are retained.

CCP divides the training-calibration pool into five folds, with each fold used once as the calibration set and the remaining folds used for model training, and the prediction set is constructed by aggregating the results from each fold. The performance of conformal prediction was evaluated using reliability metrics, including empirical coverage, average prediction set size, singleton rate, singleton accuracy, and null set rate. These metrics quantify the validity, efficiency, and stability of the prediction sets.

### 2.5 Uncertainty-aware personalized risk stratification

To improve clinical interpretability, uncertainty information derived from the CP is incorporated into the risk assessment framework. Prediction uncertainty was quantified based on the size of the CP prediction set Γ(x). A single prediction set (|Γ| = 1) was interpreted as a deterministic prediction, whereas a prediction set containing two categories (|Γ| = 2) was considered as an uncertain prediction. The predicted risk probabilities are combined with the corresponding uncertainty indicators to form a two-dimensional decision space. Individuals are categorised into four risk-certainty groups: high certainty-high risk, high certainty-low risk, low certainty-high risk, and low certainty-low risk. The stratification framework aims to identify individuals whose predictions may be unstable or underestimated under traditional risk assessment. Individualised diagnostic decision-making is supported by distinguishing between confident predictions and uncertainty dominated cases.

## 3 Results

### 3.1 Predictive performance of baseline models

In the retention test set, LR and RF showed strong discriminative performance in CAD prediction (Table 1). The accuracy of the RF model was slightly higher than that of LR (0.850 vs. 0.833). The main difference between the two models is the balance between sensitivity and specificity; LR has a higher sensitivity (0.786 vs. 0.750), while RF has a higher specificity (0.938 vs. 0.875).

**Table 1:** Baseline model performance.

Model	ACC	AUC	Sens	Spec	ECE(q10)	MCE(q10)	Brier
LR	0.833	0.953	0.786	0.875	0.097	0.389	0.089
RF	0.850	0.951	0.750	0.938	0.140	0.341	0.102

ACC, accuracy; AUC, area under the receiver operating characteristic curve; Sens, sensitivity; Spec, specificity; ECE, expected calibration error; MCE, maximum calibration error; Brier, Brier score.

**Figure 1:**
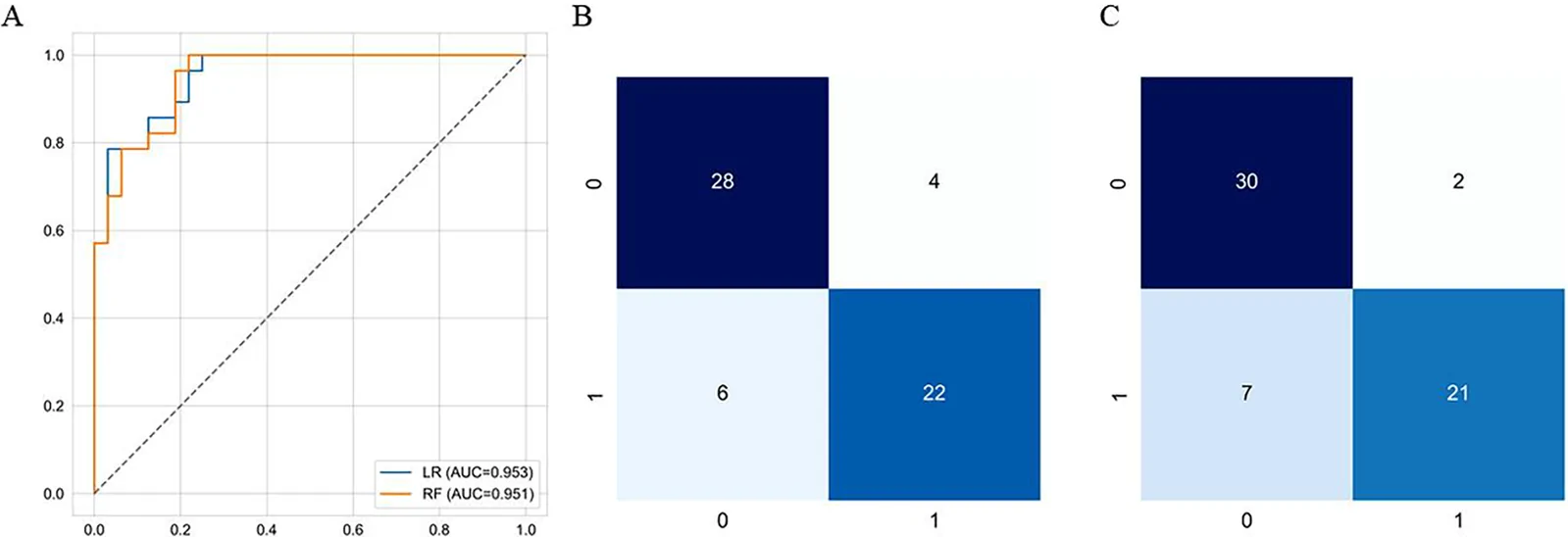
Predictive performance of baseline models. **(A)** ROC curves for LR and RF models; *x*-axis: 1-specificity (false positive rate); *y*-axis: sensitivity (true positive rate). **(B)** Confusion matrix of logistic regression; *x*-axis: predicted class; *y*-axis: actual class. **(C)** Confusion matrix of random forest; *x*-axis: predicted class; *y*-axis: actual class.

The receiver operating characteristic curves for the two models were nearly overlapping (Figure 1A), indicating comparable sorting ability. The area under the curve (AUC) was 0.953 for LR and 0.951 for RF. The corresponding confusion matrices are shown in Figures 1B,1C. The accuracy of 0.83 for LR included 4 false positives and 6 false negatives. In contrast, RF slightly improved the overall accuracy to 0.85 (TN = 30, TP = 21). Although there was a slight increase in false negatives (FN = 7), the number of false positives was reduced to two.

### 3.2 Feature relationships and model interpretation

**Figure 2:**
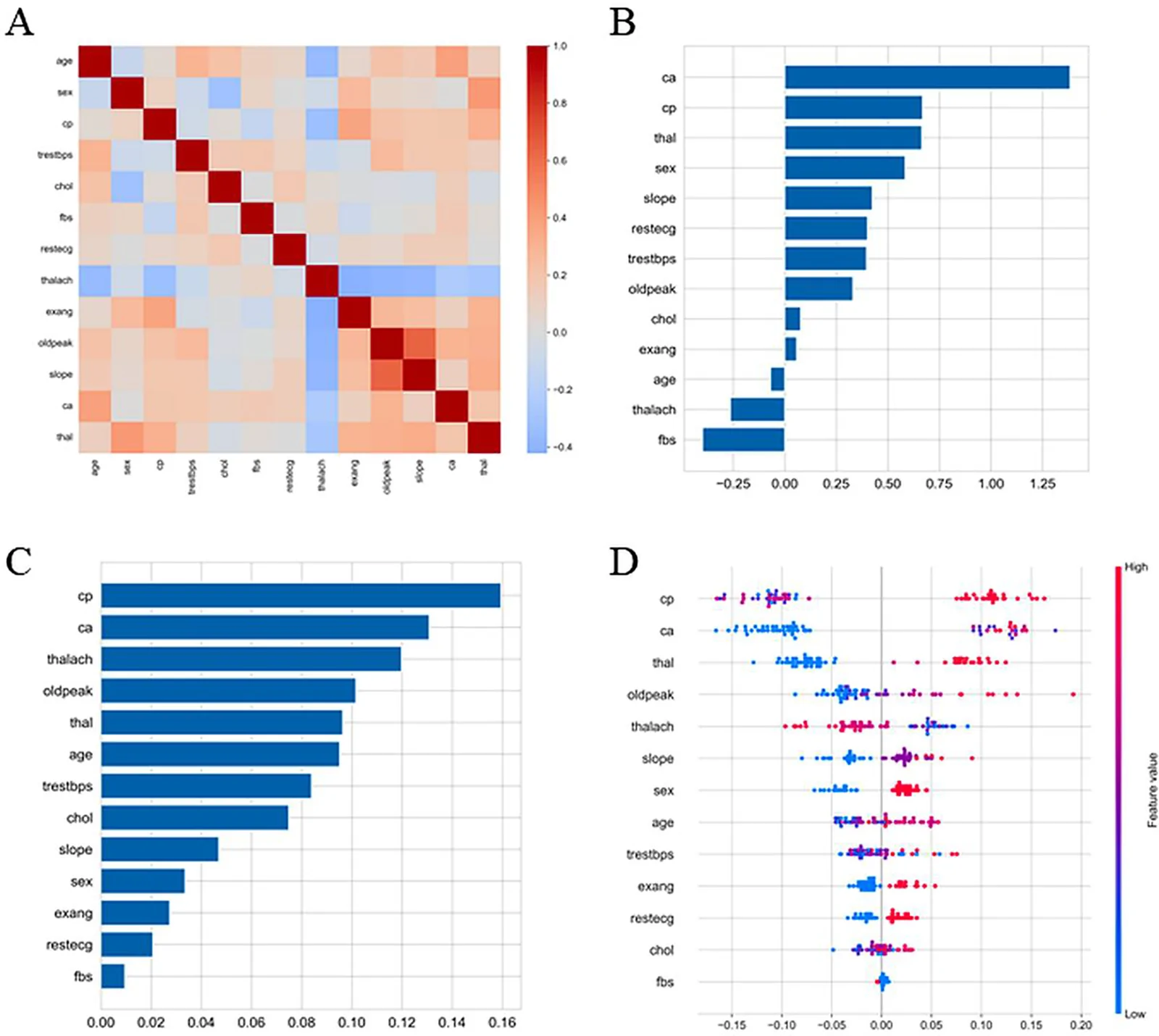
Feature relationships and model interpretation. **(A)** Correlation heatmap of clinical predictors. **(B)** Logistic regression coefficients; *x*-axis: coefficient values; *y*-axis: clinical predictors. **(C)** Random forest feature importance (Gini); *x*-axis: feature importance; *y*-axis: clinical predictors. **(D)** Random forest SHAP summary plot; *x*-axis: SHAP values; *y*-axis: clinical predictors.

Relationships between clinical predictors were explored using correlation heat maps (Figure 2A). Pairwise correlations between most variables were weak, suggesting limited multicollinearity in the set of predictors. Moderate positive correlations were observed between several variables (thalach, oldpeak, and slope), which is consistent with known physiological relationships.

Figure 2B shows the coefficient estimates from the LR model. The most influential predictors included ca, cp, and thal, all of which were positively associated with CAD risk. In contrast, the coefficients for fbs and thalach were negative. Characteristic attributions derived from the RF model yielded a similar ranking pattern. gini significance identified cp, ca, thal, oldpeak, and thalach as the most influential predictors (Figure 2C), demonstrating their central role in CAD forecasting. Further interpretation using SHAP analysis confirmed that cp, ca and thal contributed the most to the model output (Figure 2D). These findings suggest that the correlation of features is consistent across models. Considering the slightly superior classification performance of RF and its compatibility with ensemble-based uncertainty quantification, RF was chosen as the base learner for CP.

### 3.3 Performance of conformal prediction under different aggregation strategies

Overall predictive performance under different aggregation rules is reported in Table 2. With the miscoverage level set to *α* = 0.10, the union aggregation yielded complete empirical coverage (1.000) for both MSCP and CCP. This conservative behaviour was accompanied by larger prediction sets, reflected by average set sizes of 1.517 and 1.533, respectively. By comparison, the intersection rule resulted in the most compact prediction sets (1.017 vs. 0.983) and the highest proportions of singleton predictions (0.950 vs. 0.983), although empirical coverage decreased to 0.867 and 0.800. The vote aggregation displayed intermediate behaviour, maintaining coverage levels close to the theoretical guarantee (0.967 vs. 0.950) while keeping the prediction sets relatively small (1.317).

**Table 2:** Conformal aggregation comparison.

Method	Aggregation	Coverage	Avg set size	Singleton accuracy	Size singleton rate	Empty rate
MSCP	Vote	0.967	1.317	0.951	0.683	0.0
	Union	1.000	1.517	1.000	0.483	0.0
	Intersection	0.867	1.017	0.877	0.950	0.017
CCP	Vote	0.950	1.317	0.927	0.683	0
	Union	1.000	1.533	1.000	0.467	0
	Intersection	0.800	0.983	0.814	0.983	0.017

Avg Set Size: Average set size, mean number of labels per prediction set.

Singleton accuracy: accuracy when the prediction set contains a single label.

Singleton rate: proportion of prediction sets with a single label.

Empty rate: proportion of empty prediction sets (ideally 0).

**Figure 3:**
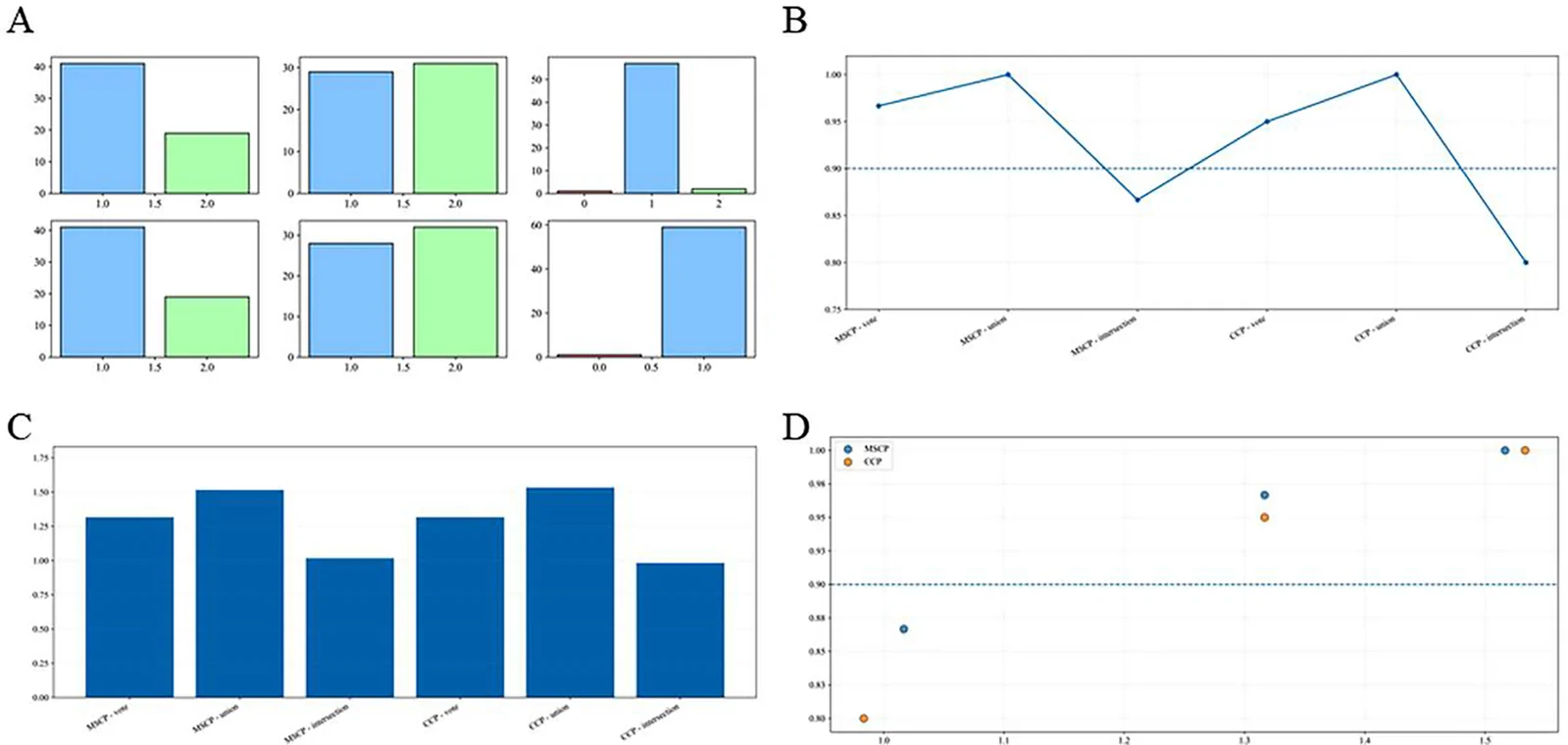
Performance comparison of conformal prediction methods. **(A)** Distribution of prediction set sizes; *x*-axis: prediction set size; *y*-axis: frequency. **(B)** Empirical coverage across methods; *x*-axis: conformal prediction methods; *y*-axis: empirical coverage. **(C)** Prediction efficiency; *x*-axis: conformal prediction methods; *y*-axis: prediction efficiency. **(D)** Coverage–efficiency trade-off. *x*-axis: prediction efficiency; *y*-axis: empirical coverage.

The distribution of prediction set sizes is shown in Figure 3A, where individual predictions dominate under the intersection rule, while the union tends to produce prediction sets containing both labels. Figure 3B illustrates the empirical coverage of the various methods, showing that the joint aggregation consistently exceeds the nominal level (1 − *α* = 0.90), while the intersection aggregation is below this threshold. Figure 3C summarises the differences in prediction efficiency, where the intersection aggregation method produces the smallest set, while the joint aggregation produces a larger set. The relationship between reliability and efficiency is further illustrated in Figure 3D, where voting aggregation occupies a region that combines relatively high coverage with moderate set sizes. Other experiments using other values of *α* demonstrate a similar pattern (Supplementary Table 2). Both corner-preserving methods maintain competitive predictive validity. Compared to CCP, MSCP has slightly higher empirical coverage and improved singleton accuracy. These results suggest a more stable balance between reliability and predictive efficiency under the MSCP model.

### 3.4 Performance of the RF–MSCP model

Based on the comparative results presented in Secs. 3.2 and 3.3, the RF and MSCP model were integrated to construct the final uncertainty-aware prediction model. The performance of the RF–MSCP model under different significance levels is summarized in Table 3. At *α* = 0.10, the empirical coverage reached 0.944 ± 0.014, exceeding the nominal level of 0.90. The average prediction set size was 1.279 ± 0.034, with a singleton prediction rate of 0.721 ± 0.034. Class-conditional coverage remained high for both categories (Class 0: 0.967 ± 0.009; Class 1: 0.919 ± 0.025). When the significance level increased to *α* = 0.15, empirical coverage was 0.884 ± 0.020, remaining above the nominal level (0.85). The average prediction set size decreased to 1.101 ± 0.043, while the singleton rate increased to 0.899 ± 0.043. Class-conditional coverage remained comparable between the two classes (Class 0: 0.927 ± 0.020; Class 1: 0.836 ± 0.025).

**Table 3:** RF–MSCP performance.

alpha	Nominal coverage	Coverage (mean ± CI)	Avg set size (mean ± CI)	Singleton rate (mean ± CI)	Class0 cov (mean ± CI)	Class1 cov (mean ± CI)
0.1	0.9	0.944 ± 0.014	1.279 ± 0.034	0.721 ± 0.034	0.967 ± 0.009	0.919 ± 0.025
0.15	0.85	0.884 ± 0.020	1.101 ± 0.043	0.899 ± 0.043	0.927 ± 0.020	0.836 ± 0.025

Nominal coverage: target coverage level specified *a priori* (1 − *α*).

**Figure 4:**
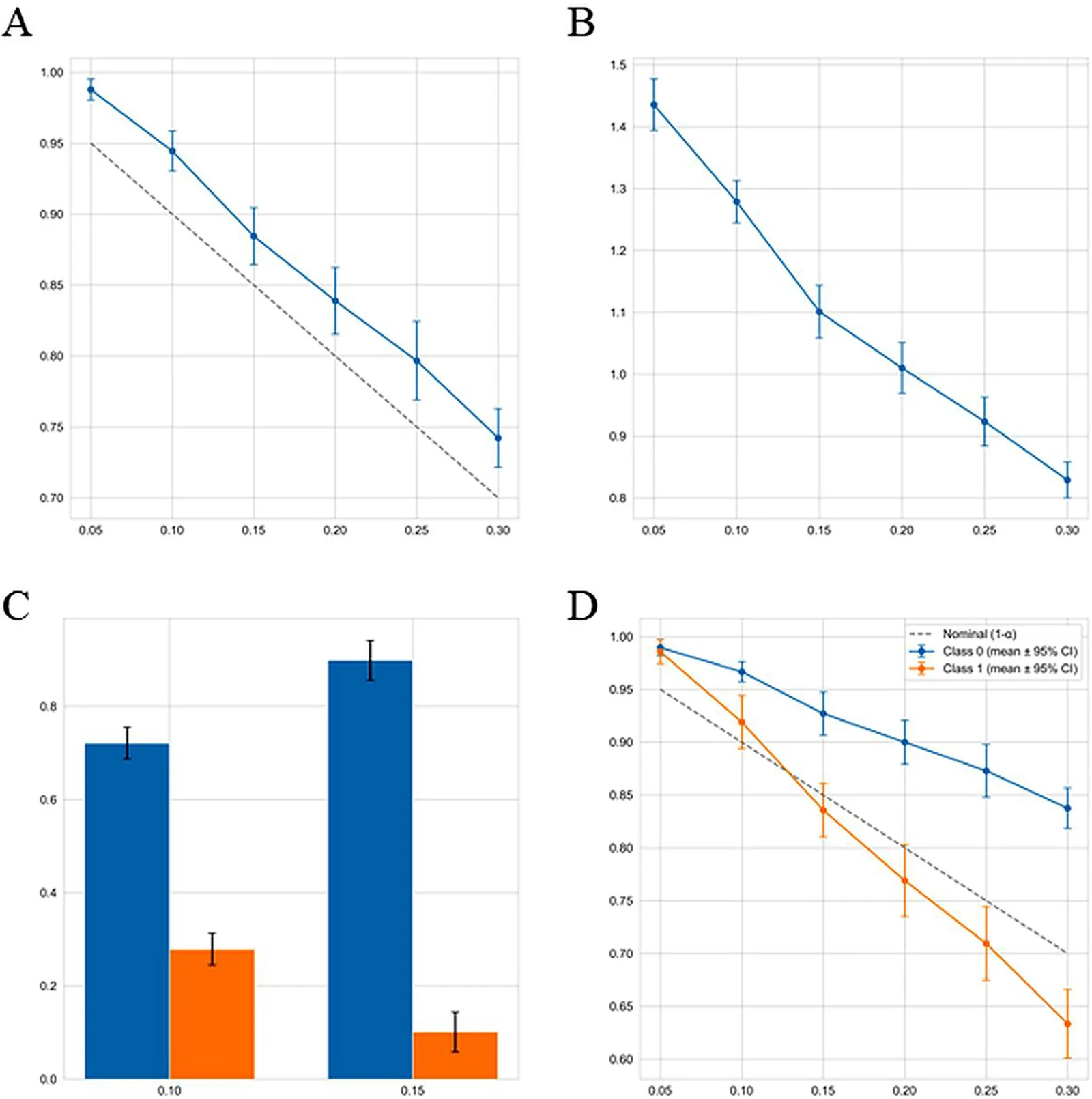
Performance of the RF–MSCP model. **(A)** Empirical coverage across *α* values; *x*-axis: significance level (*α*); *y*-axis: empirical coverage. **(B)** Prediction set across *α* values; *x*-axis: significance level (*α*); *y*-axis: prediction set size. **(C)** Distribution of prediction set sizes; *x*-axis: prediction set size; *y*-axis: frequency. **(D)** Class-conditional coverage; *x*-axis: class; *y*-axis: coverage rate.

The empirical coverage plots for different values of *α* show consistently above the nominal level (1 − *α*), indicating stable prediction in repeated experiments (Figure 4A). The relationship between the significance level and prediction efficiency is shown in Figure 4B. As *α* increases, the average prediction set size monotonically decreases. The distribution of prediction set sizes is shown in Figure 4C. Single prediction sets (|Γ| = 1) predominate, while prediction sets containing two labels (|Γ| = 2) occur less frequently. Figure 4D shows the conditional coverage of the categories for different *α* values. The coverage of both categories is close to the nominal level and does not show any serious imbalance.

### 3.5 Integration of risk prediction and uncertainty quantification

**Figure 5:**
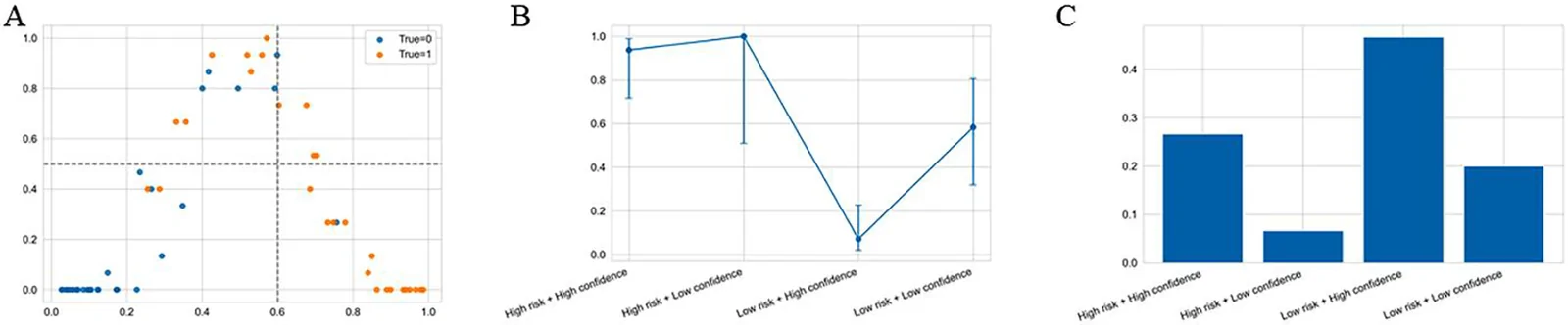
Integration of risk prediction and uncertainty. **(A)** Joint distribution of predicted risk and uncertainty; *x*-axis: predicted risk; *y*-axis: uncertainty score. **(B)** Event rates across risk–confidence groups; *x*-axis: risk–confidence groups; *y*-axis: event rate. **(C)** Population distribution of risk–confidence groups; *x*-axis: risk–confidence groups; *y*-axis: population proportion.

The joint distribution of predicted risk probability and uncertainty scores is shown in Figure 5A. In general, patients who had an event tended to be located in the high-risk region, whereas patients who did not have an event were clustered in the low-risk region. In addition to the overall distinction, uncertainty further differentiated the predictions within the two risk strata. In this cohort, uncertainty was mainly concentrated among patients with intermediate predicted risks (uncertain rate 60%), suggesting that CP-derived uncertainty was associated with ambiguous prediction patterns rather than simply reflecting estimated disease probability (Supplementary Table 3). The four risk confidence groups showed significant differences in the observed results (Figure 5B). Regardless of the confidence level, event rates remained high in the high-risk group. In contrast, in the low-risk group, event rates were substantially higher in low-confidence patients than in high-confidence patients. The distribution of the population in these groups is shown in Figure 5C. Nearly half of the cohort was categorised as low-risk with high confidence, while one-fifth of the cohort was in the low-risk but low-confidence group. Despite being categorised as low risk, this subgroup had a disproportionate number of incidents. For example, one representative individual in the test set had a predicted risk probability of 0.36 based on the RF model, which would typically be interpreted as low-to-moderate risk. However, this case exhibited a high uncertainty score (0.67) due to frequent assignment of ambiguous prediction sets across repeated CPs, and was therefore classified into the “low-risk with low certainty” group. Notably, the true outcome for this individual was positive, illustrating that the CP-based framework can flag potentially unreliable predictions even when the estimated risk is relatively low (Supplementary Table 4). Detailed results are shown in Table 4.The event rate for high-risk high-confidence patients was 0.94 (95% CI: 0.72–0.99) compared with 0.07 (0.02–0.23) for low-risk low-confidence patients. In contrast, low confidence low risk patients had a significantly higher event rate of 0.58 (0.32–0.81), close to the high risk group.

**Table 4:** Risk–confidence groups performance.

Risk–confidence quadrant	*n* (%)	Observed events (*n*)	Event rate (95% CI)	Mean predicted risk	Mean uncertainty score
High risk + high	16 (26.7)	15	0.94 (0.72–0.99)	0.86	0.10
High risk + low	4 (6.7)	4	1.00 (0.51–1.00)	0.67	0.63
Low risk + high	28 (46.7)	2	0.07 (0.02–0.23)	0.13	0.08
Low risk + low	12 (20.0)	7	0.58 (0.32–0.81)	0.48	0.85

Calibration performance is summarized in Table 5. After CP, both ECE and MCE decreased (0.085 to 0.067 and 0.187 to 0.071, respectively). While the Brier score increased slightly (0.106 to 0.117), suggesting that the improvement in calibration performance came at the cost of a decrease in sharpness. Overall, uncertainty quantification refined risk stratification by identifying a subset of nominally low-risk patients with substantially elevated event rates.

**Table 5:** Calibration metrics.

Stage	Aggregation	Alpha	ECE(q10)	MCE(q10)	Brier
Before CP (baseline prob)	—	0.1	0.085	0.187	0.106
After CP (set->point prob)	Vote	0.1	0.067	0.071	0.117

## 4 Discussion

In this study, we developed an uncertainty-aware RF–MSCP model by integrating RF with MSCP to enhance individualized risk prediction for CAD in a small-sample setting. The significant findings may suggest that while both logistic regression and random forest showed excellent discriminative ability, the incorporation of CP enabled explicit quantification of predictive uncertainty under finite-sample guarantees. It is shown that while both logistic regression and random forests exhibit excellent discriminative power, the introduction of CP enables our model to explicitly quantify prediction uncertainty with the assurance of limited samples. Results showed that individuals categorised as low risk with low confidence had significantly higher event rates. The RF-MSCP model improves the robustness of model predictions by simultaneously demonstrating risk and uncertainty, which will ultimately enhance the clinical utility of coronary artery disease prediction.

The baseline models in this study demonstrated excellent discriminative power, with AUC values exceeding 0.95, which is a level commonly achieved by machine learning algorithms in current studies. In recent years, there has been an increase in the number of highly discriminative models in cardiovascular risk prediction studies (47–50). However, these models often focus solely on AUC, whilst neglecting the assessment of model calibration and uncertainty (51–53). This imbalance may lead to excessive or insufficient inappropriate clinical decision-making that fails to convince clinicians and affects the translation of machine learning models into routine clinical practice in coronary heart disease (54). Our findings align with this perspective: traditional models with high discriminatory power do not necessarily imply predictive accuracy, underscoring the importance of incorporating uncertainty into clinical risk modelling.

Secondly, this study introduces CP to construct a principled framework for uncertainty quantification, so that the model can guarantee coverage under minimal assumptions while maintaining high confidence and coverage in the face of data with different distributions, and remain robust in the face of potential distributional biases (27, 55). Within the RF–MSCP framework, different aggregation strategies revealed a fundamental trade-off between validity and efficiency. The union strategy maximized coverage but resulted in larger prediction sets, reducing efficiency. In contrast, intersection-based aggregation improved efficiency but could be overly conservative (56). The voting-based strategy employed in our study strikes a favourable balance, maintaining reliable coverage whilst generating more informative prediction sets (57). The empirical coverage of the RF-MSCP model consistently exceeds the nominal level at multiple levels of significance, reflecting a manageable trade-off between reliability and determinism. The calibration analyses further emphasise the inherent trade-off between reliability and sharpness in probabilistic prediction. While conformal adjustment improves coverage and reliability, it is accompanied by a modest increase in Brier scores, reflecting a decrease in sharpness (43). This trade-off has been widely discussed in prior studies, and our findings support the view that incorporating uncertainty quantification may slightly compromise point prediction accuracy but substantially enhances reliability and interpretability (58).

From a clinical perspective, integrating uncertainty into CAD risk prediction carries important implications. For patients with low predictive confidence, outputting both risk and confidence yields a more cautious result rather than a single probability estimate, thus reducing the risk of misclassification (59). The dual prediction of the RF-MSCP model helps clinicians to weigh the level of risk against the confidence of the prediction, thus facilitating shared decision-making. Similar strategies have been explored in other areas, such as in the automated classification of skin cancer, where the model prompts physicians to make comprehensive judgements by labelling them “Incorrect, Correct and Certain” (24). Moreover, the tunable nature of CP allows adjustment of the significance level according to clinical priorities, offering flexibility in balancing sensitivity and specificity across different scenarios. This adaptation is particularly prominent in clinical scenarios with different emphasis on false positives or false negatives (27, 60). In addition, RF-MSCP models are advantageous in small sample scenarios. When data are limited, traditional machine learning models often suffer from overfitting and unstable probability estimates (61). In contrast, CP does not rely on distributional assumptions and guarantees validity in limited samples, making it ideal for clinical settings with small to medium data volumes (43). By combining RF with MSCP, the proposed approach achieves both strong predictive performance and statistically rigorous uncertainty quantification, offering a practical solution for real-world applications. The relatively high proportion of low-risk patients with low confidence should be interpreted in the context of the two-dimensional risk–confidence framework rather than as evidence of model failure. Although uncertainty was mainly concentrated among patients with intermediate predicted risks, a subset of patients classified as low risk still exhibited insufficient predictive confidence and a higher observed event rate, highlighting the additional value of CP beyond probability-based risk stratification.

Several limitations should be recognised. Firstly, the current RF-MSCP model is constructed on the basis of a clinical variable set; incorporation of additional data sources such as imaging or biomarkers may further enhance predictive performance and uncertainty estimates. Second, although CP provides marginal coverage guarantees under the exchangeability assumption, this guarantee may be affected when distribution shifts occur between calibration and deployment populations. Differences in patient characteristics, disease prevalence, or clinical settings may influence uncertainty estimation, which may have implications for heterogeneous clinical populations (62). In addition, this study was based on a relatively small single-centre dataset (*N* = 297), which may limit the generalisability of the findings. The high AUC values observed in the internal validation may reflect a degree of overfitting and should therefore be interpreted with caution. Finally, although the prediction set has been internally validated, further external validation in larger and more diverse patient cohorts is required before clinical application can be considered.

In conclusion, this study demonstrates that combining CP with machine learning models enables reliable and interpretable uncertainty quantification in coronary heart disease risk prediction. The proposed RF-MSCP model provides a clinically meaningful assessment of uncertainty while maintaining predictive risk. By identifying high-risk individuals vs. potentially at-risk individuals in low-risk groups, the model is expected to improve the assessment of CAD risk stratification and support more informed and nuanced clinical decision making.

## 5 Conclusion

This study shows that combining CP with machine learning models enables effective uncertainty quantification in CAD risk prediction. The proposed RF-MSCP model maintains a high level of prediction performance while ensuring effective coverage and uncertainty estimation with a small sample size. By identifying hidden risk individuals between high-risk individuals and low-risk groups, the model is expected to enhance the level of risk stratification, thus further supporting clinical translational applications and facilitating more informed clinical decisions.

## Data Availability

The original contributions presented in the study are included in the article/Supplementary Material, further inquiries can be directed to the corresponding author/s.
