# Supplementary material for: Integrating conformal prediction with machine learning for uncertainty-aware risk stratification in coronary artery disease
Source: Front Cardiovasc Med. 2026 Sep 3;13:1857390. doi: 10.3389/fcvm.2026.1857390 (PMC13581787; doi:10.3389/fcvm.2026.1857390)
Supplement: Supplementary file 1 [file Table2.docx]

Supplementary Table 2

| Alpha | Nominal Coverage | Empirical Coverage | Avg Set Size | Singleton Rate | Singleton Accuracy |
| --- | --- | --- | --- | --- | --- |
| 0.05 | 0.95 | 1.000 | 1.483 | 0.517 | 1.000 |
| 0.1 | 0.9 | 0.967 | 1.317 | 0.683 | 0.951 |
| 0.2 | 0.8 | 0.833 | 1.017 | 0.983 | 0.831 |
