# Supplementary material for: Integrating conformal prediction with machine learning for uncertainty-aware risk stratification in coronary artery disease
Source: Front Cardiovasc Med. 2026 Sep 3;13:1857390. doi: 10.3389/fcvm.2026.1857390 (PMC13581787; doi:10.3389/fcvm.2026.1857390)
Supplement: Supplementary file 2 [file Table3.docx]

Supplementary Table 3

| RF_risk_prob | U_score | Risk_group | quadrant | Y_true |
| --- | --- | --- | --- | --- |
| 0.356 | 0.667 | Low risk | Low risk+Low confidence | 1 |
