# Supplementary material for: Integrating conformal prediction with machine learning for uncertainty-aware risk stratification in coronary artery disease
Source: Front Cardiovasc Med. 2026 Sep 3;13:1857390. doi: 10.3389/fcvm.2026.1857390 (PMC13581787; doi:10.3389/fcvm.2026.1857390)
Supplement: Supplementary file 3 [file Table1.doc]

Supplementary Table 1

| Abbreviation | Full Name | Description |
| --- | --- | --- |
| age | Age | Age in years |
| sex | Sex | Gender of patient (0 = female, 1 = male) |
| cp | Chest Pain Type | Type of chest pain: 1 = typical angina, 2 = atypical angina, 3 = non-anginal pain, 4 = asymptomatic |
| trestbps | Resting Blood Pressure | Resting blood pressure measured in mmHg |
| chol | Serum Cholesterol | Serum cholesterol level in mg/dl |
| fbs | Fasting Blood Sugar | Fasting blood sugar > 120 mg/dl (1 = true, 0 = false) |
| restecg | Resting Electrocardiographic Results | Results of resting electrocardiogram |
| thalach | Maximum Heart Rate Achieved | Maximum heart rate during exercise |
| exang | Exercise Induced Angina | Exercise-induced angina (1 = yes, 0 = no) |
| oldpeak | ST Depression | ST depression induced by exercise relative to rest |
| slope | Slope of Peak Exercise ST Segment | ST segment slope: 1 = upsloping, 2 = flat, 3 = downsloping |
| ca | Number of Major Vessels | Number of major vessels colored by fluoroscopy (0–3) |
| thal | Thalassemia | Blood disorder type: 3 = normal, 6 = fixed defect, 7 = reversible defect (often recoded as 0/1/2) |
